# Clinical characteristics and predictors of severe toxicity following acute rotundine poisoning: A retrospective study of 37 patients in Vietnam

**DOI:** 10.1016/j.toxrep.2026.102312

**Published:** 2026-07-11

**Authors:** Nguyen Dang Duc, Lam Nguyen Hong Khanh, Nguyen Phuong Sinh, Le Thi Dieu Hien

**Affiliations:** a Poison Control Center, Bach Mai Hospital, Hanoi, Viet Nam; b Vinh University, Nghe An, Viet Nam; c Thai Nguyen University of Medicine and Pharmacy, Thai Nguyen, Viet Nam; d Hai Phong University of Medicine and Pharmacy, Hai Phong, Viet Nam

**Keywords:** Acute poisoning, Glasgow Coma Scale, Mechanical ventilation, QTc prolongation, Rotundine, Toxicity

## Abstract

**Background:**

Rotundine (L-tetrahydropalmatine) is widely used in Vietnam as an over-the-counter sedative and sleep aid. However, clinical data regarding acute Rotundine poisoning remain scarce. This study aimed to characterize the clinical manifestations, management, outcomes, and predictors of severe toxicity in patients with acute Rotundine poisoning.

**Methods:**

We conducted a retrospective observational study of consecutive patients with acute Rotundine poisoning admitted to the Emergency and Poison Control Department, Thai Nguyen University of Medicine and Pharmacy Hospital, Vietnam, between April 2024 and June 2026. Demographic characteristics, clinical manifestations, laboratory findings, electrocardiographic abnormalities, treatment, and outcomes were analyzed.

**Results:**

Thirty-seven patients were included. The median age was 27 years (IQR 21–41.5), and 56.8% were female. Central nervous system depression was the predominant clinical manifestation, with severe impairment of consciousness (GCS <8) occurring in 5.4% of patients. QTc prolongation (≥440 ms) was observed in 16.2%. Four patients (10.8%) required mechanical ventilation and intensive care admission. Admission GCS demonstrated excellent predictive performance for mechanical ventilation (AUC 0.989, 95% CI 0.959–1.000), with an optimal cutoff value of ≤ 12 (sensitivity 100%, specificity 97.0%). No deaths occurred.

**Conclusions:**

Acute Rotundine poisoning generally has a favorable prognosis but may occasionally cause severe central nervous system depression requiring intensive care and mechanical ventilation. Admission GCS may serve as a simple bedside predictor of severe toxicity, while electrocardiographic monitoring should be considered because QTc prolongation is relatively common.

## Highlights

• Acute Rotundine poisoning predominantly causes central nervous system depression.
• Admission Glasgow Coma Scale accurately predicts the need for mechanical ventilation.
• Mild QTc prolongation is relatively common after acute Rotundine overdose.
• Most patients recover with supportive care despite occasional severe toxicity.

## Graphical Abstract

**Figure:**
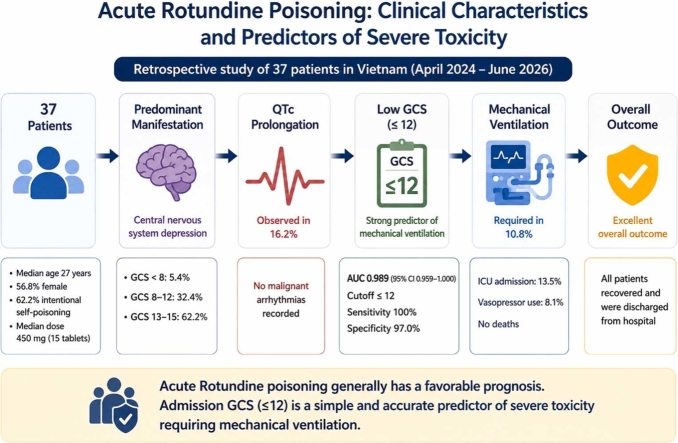

## 1 Introduction

Rotundine (L-tetrahydropalmatine, L-THP) is a naturally occurring isoquinoline alkaloid isolated from several species of the genus *Stephania* and other medicinal plants widely used in traditional Asian medicine [1], [2], [3], [4]. Owing to its sedative, anxiolytic, analgesic, and sleep-promoting properties, Rotundine has been extensively utilized in several Asian countries for the management of insomnia, anxiety, and mild pain disorders [3], [4]. In Vietnam, Rotundine is commonly available as an over-the-counter medication, typically formulated as 30-mg tablets, and is frequently used for self-medication because of its perceived safety and easy accessibility.

Pharmacokinetic studies have demonstrated that L-tetrahydropalmatine is rapidly absorbed following oral administration, undergoes extensive hepatic metabolism, and is primarily eliminated through hepatic biotransformation. Peak plasma concentrations are generally achieved within approximately 1–2 h after administration, although substantial interindividual variability has been reported. Because metabolism predominantly occurs in the liver, excessive exposure may increase the risk of prolonged central nervous system depression and other dose-related adverse effects [4], [5], [10].

The pharmacological effects of Rotundine are primarily mediated through modulation of central dopaminergic neurotransmission. Experimental studies have demonstrated that L-THP acts as an antagonist at dopamine D1 and D2 receptors and may additionally influence serotonergic, adrenergic, and GABAergic pathways [3], [5], [6]. These mechanisms are believed to underlie its sedative and hypnotic effects. More recently, L-THP has attracted research interest as a potential therapeutic agent for substance use disorders and neuropsychiatric conditions because of its ability to attenuate reward-related dopaminergic signaling [1], [2], [7], [8], [9]. In addition to dopaminergic receptor antagonism, experimental evidence suggests that L-THP may modulate γ-aminobutyric acid (GABA), serotonergic, and adrenergic neurotransmission, thereby enhancing its sedative and hypnotic properties. These multiple pharmacodynamic effects probably explain the progressive impairment of consciousness observed following overdose [3], [4], [5], [6], [7].

Although Rotundine is generally considered safe at therapeutic doses, adverse effects including excessive sedation, dizziness, hypotension, and hepatotoxicity have been reported [10], [11], [12], [13]. Although therapeutic doses of Rotundine are generally considered safe, a precise toxic dose in humans has not been established. Published reports indicate considerable interindividual variability in toxicity, with clinical severity depending not only on the ingested dose but also on drug absorption, hepatic metabolism, coexisting medical conditions, and individual susceptibility. Reported manifestations of overdose include drowsiness, somnolence, dizziness, hypotension, respiratory depression, coma, and occasionally electrocardiographic abnormalities [4], [10], [11], [12], [13]. Information regarding the clinical manifestations of acute Rotundine overdose remains limited. Most available literature focuses on pharmacological properties, animal experiments, or therapeutic applications rather than acute poisoning [4], [7], [9]. Consequently, the spectrum of toxicity associated with Rotundine overdose in humans remains poorly characterized. Several case reports and pharmacovigilance studies have suggested that excessive exposure to tetrahydropalmatine-containing preparations may occasionally result in severe neurological depression; however, systematic clinical descriptions of acute overdose remain scarce.

In particular, there is limited information regarding the neurological manifestations, cardiovascular effects, electrocardiographic abnormalities, laboratory disturbances, and clinical outcomes of acute Rotundine poisoning. Drug-induced cardiac conduction abnormalities, including QTc interval prolongation, have become increasingly recognized as clinically important adverse events associated with numerous centrally acting medications [14], [15], [16]. Whether similar electrophysiological disturbances occur following Rotundine overdose has not been adequately investigated. Despite the widespread use of Rotundine throughout Vietnam, systematic clinical studies describing acute poisoning remain extremely limited. Most available publications focus on pharmacological properties, experimental models, or therapeutic applications rather than the clinical presentation and management of overdose. Consequently, evidence regarding predictors of severe toxicity and clinically useful prognostic indicators remains insufficient.

Given the widespread availability of Rotundine in Vietnam and the scarcity of clinical toxicology data, further characterization of overdose incidents is warranted. Therefore, this study aimed to characterize the clinical features, electrocardiographic abnormalities, management, clinical outcomes, and predictors of severe toxicity in patients with acute Rotundine poisoning.

## 2 Methods

This retrospective observational study was conducted and is reported in accordance with the Strengthening the Reporting of Observational Studies in Epidemiology (STROBE) statement. The study was performed at the Emergency and Poison Control Department, Thai Nguyen University of Medicine and Pharmacy Hospital, Thai Nguyen, Vietnam.

Consecutive patients admitted between April 2024 and June 2026 with a diagnosis of acute Rotundine poisoning were screened for eligibility. Eligible patients fulfilled all inclusion criteria and none of the exclusion criteria.

Inclusion criteria:

• Age ≥ 18 years.

• History of acute Rotundine ingestion. • Clinical manifestations consistent with acute poisoning. • Sufficient medical records available for review.

Exclusion criteria:

• Co-ingestion of other medications or toxic substances confirmed by history or medical records. • Incomplete clinical data.

The following variables were extracted from medical records: age, sex, medical history, suicidal intent, estimated dose ingested, time from ingestion to hospital admission, level of consciousness, pupil size, heart rate, respiratory rate, blood pressure, QRS duration, QTc interval, hemoglobin, hematocrit, white blood cell count, platelet count, lymphocyte count, urea, creatinine, AST, ALT, creatine kinase, troponin, sodium, potassium, and chloride. QTc prolongation was defined as a corrected QT interval ≥ 440 ms. Clinical outcomes included intensive care unit admission, mechanical ventilation, vasopressor requirement, hospital length of stay, and in-hospital mortality.

Continuous variables were expressed as mean ± standard deviation (SD) for normally distributed data or median with interquartile range (IQR) for non-normally distributed data. Categorical variables were expressed as frequencies and percentages. Normality was assessed using the Shapiro–Wilk test. Comparisons between ventilated and non-ventilated patients were performed using the Mann–Whitney *U* test for continuous variables and Fisher's exact test for categorical variables when appropriate. Receiver operating characteristic (ROC) curve analysis was used to evaluate the predictive performance of admission Glasgow Coma Scale (GCS) for mechanical ventilation. A two-sided P value < 0.05 was considered statistically significant.

### 2.1 Ethical considerations

This study was approved by the Institutional Ethics Committee of Thai Nguyen University of Medicine and Pharmacy Hospital, Thai Nguyen, Vietnam (Approval No. TUMP−2024–04EC). Patient confidentiality was maintained throughout the study, and all data were anonymized prior to analysis.

## 3 Results

Thirty-seven patients met the inclusion criteria. Baseline demographic and poisoning characteristics are presented in Table 1.

**Table 1:** Baseline Characteristics and Poisoning Characteristics.

Variable	Value
Age (years) (median, IQR)	27 (21–41.5)
Female sex, n (%)	21 (56.8)
History of chronic disease, n (%)	17 (45.9)
Suicidal ingestion, n (%)	23 (62.2)
Estimated dose (mg)(median, IQR)	450 (300−900)
Number of tablets ingested	15 (10−30)
Time from ingestion to admission (hours) (median, IQR)	3 (2−7)

The median age was 27 years (IQR 21–41.5), and 21 patients (56.8%) were female. Seventeen patients (45.9%) had underlying chronic medical conditions. Intentional self-poisoning accounted for 23 cases (62.2%). The median estimated Rotundine dose was 450 mg (IQR 300–900), corresponding to 15 tablets.

Altered mental status represented the most clinically important manifestation of toxicity.

Drowsiness occurred in 7 patients (18.9***%)***, while somnolence was observed in 1 patient (2.7%). Severe central nervous system depression with GCS < 8 occurred in 2 patients (5.4%).

Clinical manifestations and electrocardiographic findings are summarized in Table 2.

**Table 2:** Clinical Manifestations and Electrocardiographic Findings.

Variable	n (%) or median (IQR)
Drowsiness	7 (18.9)
Somnolence	1 (2.7)
Miosis	2 (5.4)
Heart rate	71 (63.5–86.5)
Respiratory rate	20 (19−20)
Systolic blood pressure	120 (108.5–130)
Diastolic blood pressure	70 (68.5–80)
GCS score	15 (14−15)
GCS < 8	2 (5.4)
QTc interval (ms)	420 (409−436)
QTc ≥ 440 ms	6 (16.2)
QTc ≥ 450 ms	3 (8.1)

QTc prolongation, defined as QTc ≥ 440 ms, was identified in 6 patients (16.2%), including 3 patients (8.1%) with QTc ≥ 450 ms. The median QTc interval was 420 ms (IQR 409–436), and the maximum recorded QTc interval was 460 ms. No episodes of ventricular tachycardia, torsades de pointes, or sudden cardiac arrest were observed. No patient developed clinically significant conduction disturbances requiring specific antiarrhythmic treatment.

Laboratory abnormalities are summarized in Table 3.

**Table 3:** Admission Laboratory Characteristics of Patients with Acute Rotundine Poisoning.

Variable	Value (median, IQR)
Hemoglobin	135 (127.5–144.5)
Hematocrit	0.397 (0.383–0.433)
White blood cells	9.16 (7.48–11.54)
Platelets	271 (199.5–313)
Urea	4.1 (3.55–4.95)
Creatinine	70 (63.5–82.5)
AST	21 (17−26)
ALT	14 (10–20.5)
CK	87 (68.95–113)
Troponin	6.12 (3.67–10)
Sodium	140 (136−141)
Potassium	3.8 (3.55–4.0)
Chloride	103 (101−105)

Elevated AST was observed in 3 patients (8.1%), elevated ALT in 3 patients (8.1%), elevated CK in 3 patients (8.1%), and elevated troponin in 4 patients (10.8%). None of these patients demonstrated clinical evidence of acute coronary syndrome or hemodynamically significant cardiac dysfunction.

Management and Outcomes as in Table 4.

**Table 4:** Clinical Management and Outcomes of Patients with Acute Rotundine Poisoning.

Variable	n (%) or median (IQR)
ICU admission	4 (10.8)
Mechanical ventilation	4 (10.8)
Vasopressor use	1 (2.7)
Hospital stay (days) (median, IQR)	2 (2–4.5)
Death	0 (0)

Overall clinical outcomes are summarized in Table 4.

Supportive treatment was administered to all patients.

Mechanical ventilation was required in 4 patients (10.8%). ICU admission occurred in 4 patients (10.8%). Vasopressor support was used in 1 patient (2.7%).

The median length of hospital stay was 2 days (IQR: 2– 4.5).

No deaths occurred during hospitalization.

Baseline clinical and laboratory characteristics were compared between ventilated and non-ventilated patients. Detailed comparisons are presented in Table 5, demonstrating that admission GCS was the only variable significantly associated with the need for mechanical ventilation. Patients requiring mechanical ventilation had significantly lower admission GCS scores (median 8.5 vs 15, p < 0.001), whereas age, reported ingested dose, QTc interval, cardiac biomarkers, and liver enzyme concentrations did not differ significantly between groups.

**Table 5:** Comparison between ventilated and non-ventilated patients.

Variable (median, IQR)	Ventilated	Non-ventilated	P value (Mann-Whitney U)
Age	47.5 (24.25–82)	27 (20.5–40.5)	0.155
Time to hospital	7.5 (1.25–21.25)	3 (2–6.5)	0.621
Dose ingested	600 (300−915)	405 (315−450)	0.362
GCS	8.5 (6.25–10.75)	15 (14.5–15)	< 0.001
QTc	429.5 (410.25–444.25)	418 (409−435)	0.54
Troponin	9.925 (4.1775–37.67)	6.08 (3.565–9.71)	0.22
CK	118.5 (42.75–246.75)	87 (69−109)	0.883
AST	24.5 (20.5–50.25)	20 (16.5–24.5)	0.186
ALT	16 (11.25–43.25)	14 (10–20.5)	0.404

**Fig. 1:**
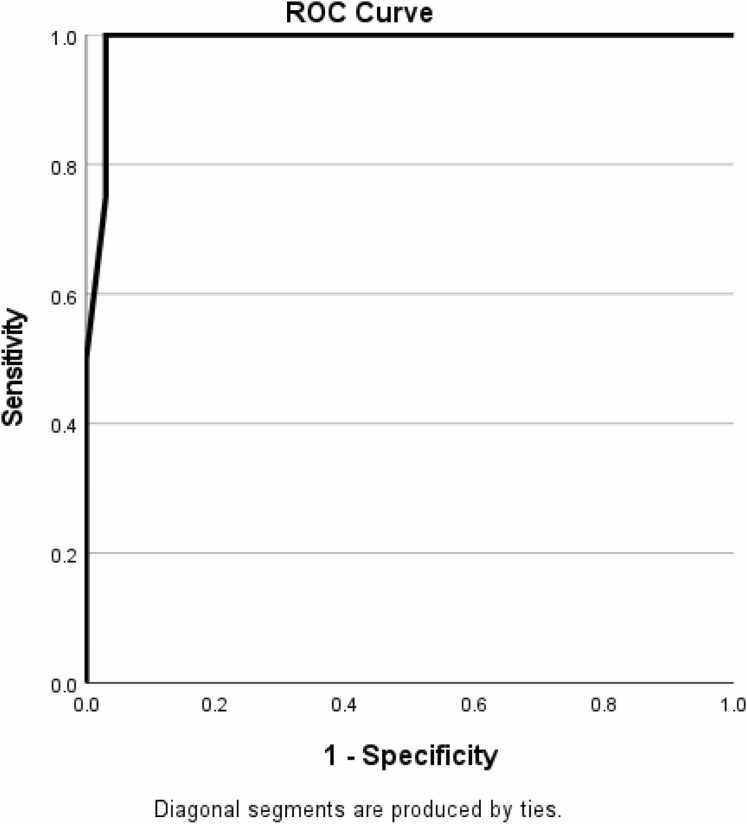
Receiver operating characteristic (ROC) curve of admission Glasgow Coma Scale (GCS) for predicting the need for mechanical ventilation in acute Rotundine poisoning. The area under the curve was 0.989 (95% CI 0.959–1.000).

Receiver operating characteristic (ROC) analysis was performed to evaluate the ability of admission Glasgow Coma Scale (GCS) to predict the need for mechanical ventilation. Admission GCS demonstrated excellent discriminatory performance, with an area under the ROC curve (AUC) of 0.989 (95% CI 0.959–1.000, p = 0.002). A GCS cutoff value of ≤ 12 provided the optimal balance between sensitivity and specificity, yielding a sensitivity of 100% and a specificity of 97.0%. The optimal cutoff value was selected according to the maximum Youden index. The ROC curve is presented in Fig. 1.

## 4 Discussion

To our knowledge, this study represents one of the largest clinical series describing acute Rotundine poisoning reported to date. The principal findings were that acute Rotundine poisoning predominantly caused central nervous system depression, while severe toxicity requiring mechanical ventilation occurred in approximately one in ten patients. In addition, mild QTc prolongation was relatively common, whereas clinically significant hepatic or cardiovascular injury was uncommon. Importantly, admission Glasgow Coma Scale demonstrated excellent performance in identifying patients at risk of mechanical ventilation. These findings provide clinically relevant evidence to support early neurological assessment and bedside risk stratification in patients with acute Rotundine poisoning.

Central nervous system depression was the predominant manifestation of toxicity in our cohort, consistent with the known pharmacological profile of L-tetrahydropalmatine. An important finding of our study was that four patients (10.8%) required mechanical ventilation. This observation suggests that acute Rotundine poisoning should not be considered uniformly benign. Although most patients experienced only mild-to-moderate toxicity and recovered with supportive care, severe overdose may result in profound central nervous system depression, loss of airway protective reflexes, and respiratory compromise requiring endotracheal intubation and ventilatory support. Notably, comparison between ventilated and non-ventilated patients demonstrated a significantly lower admission Glasgow Coma Scale (GCS) score among ventilated patients, whereas the reported ingested dose was not significantly different between the two groups. These findings suggest that early neurological assessment may be more clinically useful than estimated dose in identifying patients at risk of severe poisoning and respiratory failure. Because the accuracy of self-reported ingestion history is often limited in overdose settings, reliance on clinical evaluation, particularly the level of consciousness, may provide more reliable guidance for risk stratification and airway management decisions. Similar observations have been reported in benzodiazepine overdose, barbiturate poisoning, and other sedative-hypnotic intoxications, in which neurological examination is generally a better predictor of respiratory compromise than the reported ingested dose because overdose histories are frequently inaccurate or incomplete [9], [10], [11]. From a clinical perspective, this finding is particularly important for emergency physicians because the GCS can be assessed immediately at presentation without laboratory investigations. Early identification of patients at risk of respiratory failure may facilitate timely airway protection and intensive monitoring. The ROC analysis further supported the prognostic value of admission GCS. An AUC of 0.989 (95% CI 0.959–1.000) indicated excellent discrimination for identifying patients who subsequently required mechanical ventilation. A GCS threshold of ≤ 12 achieved 100% sensitivity and 97.0% specificity in our cohort. Because airway protection is a critical priority in poisoning management, a highly sensitive threshold may be clinically valuable for identifying patients requiring close monitoring or early airway intervention. Nevertheless, the excellent predictive performance observed in the present study should be interpreted cautiously because only four patients required mechanical ventilation. External validation using larger multicenter cohorts is required before this threshold can be recommended for routine clinical practice.

Another notable finding was the occurrence of mild electrocardiographic abnormalities in a subset of patients. QTc prolongation (QTc ≥440 ms) occurred in 16.2% of patients. However, the degree of QTc prolongation was generally modest, with no patient exhibiting a QTc interval exceeding 460 ms. Although no episodes of torsades de pointes, ventricular tachycardia, or sudden cardiac arrest were observed, these findings warrant attention because drug-induced QTc prolongation is a recognized precursor of potentially life-threatening ventricular arrhythmias [14], [15], [16]. Although the clinical significance of these mild QTc changes remains uncertain, routine electrocardiographic monitoring appears reasonable in patients with substantial overdose or impaired consciousness. The mechanisms underlying QT prolongation in Rotundine poisoning remain unclear. Excessive concentrations of centrally acting compounds may influence cardiac ion channel activity either directly or indirectly through autonomic nervous system effects. Although our data do not establish causality, the observed frequency of QTc abnormalities supports routine electrocardiographic monitoring in patients presenting with significant Rotundine overdose. Although human data regarding Rotundine-associated QTc prolongation remain extremely limited, drug-induced QTc prolongation has been well documented following overdose with several centrally acting medications. The absence of malignant ventricular arrhythmias in our cohort is reassuring; however, continuous electrocardiographic monitoring remains advisable in patients presenting with severe poisoning because clinically important arrhythmias may occur unpredictably. Compared with previous reports describing marked QTc prolongation and malignant ventricular arrhythmias associated with other sedative agents, QTc prolongation in our cohort was generally modest and was not accompanied by torsades de pointes or sudden cardiac death [14], [15], [16].

In our cohort, however, liver enzyme elevations were infrequent and clinically mild. Previous reviews have described occasional hepatotoxicity associated with prolonged exposure to tetrahydropalmatine-containing herbal products. In contrast, liver enzyme abnormalities in our patients were uncommon and clinically mild, suggesting that acute overdose predominantly affects the central nervous system rather than producing severe hepatic injury [11], [12], [13]. Mild troponin elevation was observed in four patients but was not associated with clinically significant cardiac dysfunction or arrhythmias. These findings suggest that transient myocardial stress rather than direct cardiotoxicity may explain the observed biomarker abnormalities.

The demographic characteristics of our cohort are also noteworthy. The median age was 27 years, and nearly two-thirds of cases were associated with intentional self-poisoning. The predominance of intentional self-poisoning observed in our cohort is consistent with poisoning epidemiology reported from several low- and middle-income countries, where readily accessible prescription and non-prescription medications frequently constitute the agents used in self-harm attempts. Increased public awareness and appropriate mental health interventions may therefore contribute to reducing poisoning incidence. Interestingly, the estimated ingested dose was not significantly associated with the requirement for mechanical ventilation. This finding suggests that interindividual variability in pharmacokinetics, absorption, and susceptibility to central nervous system depression may play an important role in determining clinical severity. Similar discrepancies between reported dose and clinical outcome have been observed in other pharmaceutical overdoses. These findings suggest that clinicians should prioritize clinical assessment over estimated ingestion history when determining the severity of Rotundine poisoning.

Despite the occurrence of severe poisoning in several patients, overall outcomes were favorable. No deaths occurred, and the median hospital stay was only two days. These findings suggest that supportive management remains highly effective for most patients with acute Rotundine poisoning. Early airway assessment, careful neurological monitoring, cardiovascular observation, and appropriate supportive care appear sufficient in the majority of cases. The absence of mortality in our cohort is reassuring and supports the view that prognosis is generally good when timely medical care is available. Our findings are consistent with the review by Du et al., who reported that CNS depression represents the principal pharmacological effect of L-THP, whereas severe acute overdose in humans has rarely been systematically described. Similar favorable outcomes have been reported in the limited published literature describing Rotundine exposure, suggesting that most patients recover completely with timely supportive treatment. Nevertheless, severe neurological toxicity has been described following excessive ingestion, emphasizing the importance of early recognition and appropriate airway management. From a clinical perspective, patients presenting after Rotundine overdose should undergo early neurological assessment, serial GCS evaluation, continuous electrocardiographic monitoring, and prompt airway assessment when consciousness deteriorates. Because no specific antidote is currently available, supportive care remains the cornerstone of management. Early recognition of patients at risk for respiratory failure may facilitate timely intensive care admission and improve clinical outcomes.

This study has several important strengths. First, it represents one of the largest clinical series of acute Rotundine poisoning currently available. Second, comprehensive demographic, clinical, laboratory, and electrocardiographic data were systematically analyzed. Third, the study is the first to evaluate the diagnostic performance of admission GCS for predicting mechanical ventilation in patients with acute Rotundine poisoning. Finally, this study provides one of the first clinically applicable bedside prediction models using admission GCS for identifying patients at risk of mechanical ventilation. Another strength is that consecutive patients were included, reducing selection bias and reflecting routine clinical practice.

Nevertheless, several limitations should be acknowledged. The retrospective design may have introduced information bias and limited the availability of certain clinical variables. The study was conducted at a single center, potentially reducing generalizability to other settings. Second, information regarding the exact amount ingested relied largely on patient or family reports and may therefore be subject to recall bias. Furthermore, only four patients required mechanical ventilation. Consequently, the ROC-derived cutoff value and estimates of diagnostic accuracy may be optimistic and should be validated in larger multicenter studies. In addition, the relatively small sample size limited statistical power and precluded multivariable regression analysis to identify independent predictors of severe toxicity. Finally, because serum L-tetrahydropalmatine concentrations were unavailable, correlations between blood drug concentration and clinical severity could not be assessed.

In conclusion, our findings indicate that acute Rotundine poisoning is characterized predominantly by central nervous system depression, with occasional severe cases requiring intensive care and mechanical ventilation. Electrocardiographic abnormalities, particularly QTc prolongation, were common but were not associated with malignant arrhythmias in this cohort. Although overall prognosis was favorable, clinicians should remain aware of the potential for severe neurological toxicity and cardiac conduction abnormalities following Rotundine overdose.

## 5 Conclusions

Acute Rotundine poisoning predominantly manifests as central nervous system depression and generally has a favorable prognosis with appropriate supportive care. Nevertheless, a clinically important proportion of patients may develop severe neurological impairment requiring intensive care and mechanical ventilation. Admission Glasgow Coma Scale (GCS) demonstrated excellent performance in identifying patients at risk of respiratory failure and may serve as a simple, rapid, and practical bedside tool for early risk stratification in emergency settings. Although QTc prolongation was relatively common, clinically significant ventricular arrhythmias were not observed in this cohort. Given the increasing availability and widespread use of Rotundine in Vietnam, emergency physicians and clinical toxicologists should remain vigilant for severe overdose presentations and ensure appropriate neurological and electrocardiographic monitoring. Larger prospective multicenter studies are warranted to validate these findings and to further establish evidence-based management strategies for acute Rotundine poisoning.

## CRediT authorship contribution statement

**Lam Nguyen Hong Khanh:** Methodology. **Nguyen Dang Duc:** Writing – review & editing, Writing – original draft, Supervision. **Le Thi Dieu Hien:** Resources. **Nguyen Phuong Sinh:** Investigation.

## Declaration of Competing Interest

The authors declare the following financial interests/personal relationships which may be considered as potential competing interests:Nguyen Dang Duc reports administrative support, article publishing charges, equipment, drugs, or supplies, statistical analysis, travel, and writing assistance were provided by Bach Mai Hospital. Nguyen dang duc reports a relationship with Bach Mai Hospital that includes: employment. Nguyen Dang Duc has patent #BMH_04–2024 pending to 1234567. Not conflict If there are other authors, they declare that they have no known competing financial interests or personal relationships that could have appeared to influence the work reported in this paper.

## Data Availability

The data that has been used is confidential.
